# Combined treatment of molnupiravir and favipiravir against SARS-CoV-2 infection: One + zero equals two?

**DOI:** 10.1016/j.ebiom.2021.103663

**Published:** 2021-11-09

**Authors:** Philippine Eloy, Roger Le Grand, Denis Malvy, Jérémie Guedj

**Affiliations:** a INSERM CIC-EC 1425, Hôpital Bichat Claude Bernard, 75018 Paris, France; b Université Paris-Saclay, Inserm, CEA, Center for Immunology of Viral, Auto-immune, Hematological and Bacterial diseases (IMVA-HB/IDMIT), Fontenay-aux-Roses & Le Kremlin-Bicêtre, Paris, France; c Inserm, UMR 1219, Université de Bordeaux, Bordeaux, France; d Centre Hospitalier Universitaire de Bordeaux, Bordeaux, France; e Université de Paris, IAME, INSERM, F-75018 Paris, France

The route to an effective antiviral treatment against SARS-CoV-2 has been marked by dozens of negative clinical trials, with thousands of patients across the globe enrolled in futile clinical trials. Too often, emergency has been an excuse for testing drugs with no or poor pharmacological rationale, bypassing the standard process of validations in pre-clinical *in vitro* and *in vivo* models.

Nearly 60 randomized clinical trials worldwide are registered on *clinicaltrial.gov* to evaluate the efficacy of favipiravir in SARS-CoV-2 infected patients, either as monotherapy or in combination. In most studies, the dose used was 600-800 mg BID, which is the dose used in the clinical development of favipiravir against pandemic influenza virus infection and severe influenza illness. These studies display a large heterogeneity regarding the population (inpatients and outpatients) and the primary endpoint (virological or clinical). Although some studies suggest that favipiravir could decrease the time to viral clearance in mild or moderate COVID-19 patients [1] or the time to clinical improvement [2], the retrospective aspect or the absence of randomization of most studies precludes a definite answer on favipiravir efficacy. As pointed out by the meta-analysis conducted by the COVID-NMA initiative (www.covid-nma.com), the level of evidence on favipiravir virological and clinical endpoints remains therefore low to very low.

Molnupiravir has been studied in SARS-CoV-2 infected patients as a monotherapy at the dose of 800 mg BID in outpatients and hospitalized patients, with both virological and clinical endpoints. Data from phase II/III trial showed that molnupiravir is unlikely to demonstrate a clinical benefit in hospitalized patients. In outpatients, molnupiravir reduces the risk of hospitalization or death by approximately 50 percent compared to placebo for patients with mild or moderate COVID-19 in an interim analysis of phase III study [3].

The study of Abdelnabi et al. [4] builds on previous pre-clinical studies that showed a marked activity of molnupiravir against SARS-CoV-2, with an micromolar activity *in vitro* and evidence of antiviral activity and reduced risk of transmission in several animal models (mice, hamster, ferrets) [5,6]. Determination of favipiravir antiviral activity is less well established, with an activity that ranges from 60 to ∼500 µM [7]. By thoroughly evaluating the antiviral activity of these drugs in the hamster model, alone or in combination, Abdelnabi et al. [4] demonstrate that the combination of the two drugs at suboptimal doses is synergic, and leads to a strong reduction in both total viral load and infectious virus, when treatment is administered before or very rapidly after infection. Further, they show that administration of treatment in hamsters can dramatically reduce the risk of infection to co-housed hamsters. These results are important for clinical development. They suggest that molnupiravir could be used at a lower dose, in combination with favipiravir, to increase virus mutagenesis and bring the virus close to error catastrophe. Because these drugs can be given orally and are likely less sensitive to mutations in the spike proteins [6], this combination could also be administered easily and require a less stringent virological surveillance than monoclonal antibodies.

Before this combination can be tested in clinical studies, several important questions will need to be studied in depth. In their experimental model, the synergy was achieved at doses where either drug had demonstrated an antiviral activity. At this day, favipiravir has shown an antiviral activity in the hamster model but not in larger animal models or in humans. This may be due to the fact that the rapid metabolic activity in hamster allows to test high doses of drugs. In that respect, a critical point might be therefore the complexity of favipiravir pharmacokinetics and the need to use higher doses in humans than what has been used so far to achieve such antiviral activity. Pharmacokinetic models suggested that doses of 1600 mg BID may be needed to achieve such relevant drug concentrations, which is more much larger than the dose used in most clinical trials. Therefore, pharmacokinetic and tolerance studies will be needed to assess the possibility of using such high doses in humans [8]. Safety will be particularly important given that this type of combination will primarily be used in individuals that have not yet developed a highly symptomatic infection, in post exposure prophylaxis or in the first days that follow symptom onset.

The results of Abdenabi et al. could also have important implications for other RNA viruses. Indeed, both favipiravir and molnupiravir have a panviral activity, with evidence of activity against several viruses, including influenza and hemorrhagic fever viruses [9,10]. Previous studies already showed that the combination of favipiravir and ribavirin could be synergic against Lassa virus. Their results could therefore be applied rapidly in other contexts, for which there is a desperate lack of effective antiviral treatments.

## Contributors

All authors contributed equally to this commentary.

## Declaration of Competing Interest

The authors report ongoing collaborations between their institution, INSERM, and Toyama, the manufacturer of favipiravir.
